# Understanding Duchenne muscular dystrophy-associated brain pathology

**DOI:** 10.1242/dmm.052302

**Published:** 2025-08-01

**Authors:** Minou A. T. Verhaeg, Rosanne Govaarts, Maaike van Putten

**Affiliations:** ^1^ Department of Human Genetics, Leiden University Medical Center, 2333 ZC Leiden, The Netherlands; ^2^ C.J. Gorter MRI Center, Department of Radiology, Leiden University Medical Center, 2333 ZA Leiden, The Netherlands

**Keywords:** Dystrophin, Comorbidities, DMD mouse models, Exon skipping therapy

## Abstract

The most common neuromuscular disorder, Duchenne muscular dystrophy (DMD), is caused by mutations in the *DMD* gene, resulting in a lack of dystrophin. In addition to severe and progressive muscle wasting, a subset of individuals with DMD experience, to largely varying extents, behavioural and cognitive deficits, including a lower IQ, and neurological comorbidities, such as autism spectrum disorder, obsessive compulsive disorder and attention deficit hyperactivity disorder. Neuroimaging studies in individuals with DMD have identified widespread pathology, including structural, physiological and connective alterations. DMD mouse models exhibit a number of DMD-associated behavioural traits, including anxiety, social deficits and learning disabilities, and have been used to investigate DMD brain pathology. Although there are currently no therapies to treat DMD brain pathology, genetic approaches are being developed to restore dystrophin expression. In particular, the exon skipping approach shows promise in ameliorating certain DMD-associated behavioural deficits in preclinical settings. However, the therapeutic potential of postnatal restoration of dystrophin isoforms involved in neurodevelopment is unknown. Furthermore, challenges such as low dystrophin restoration efficacy and translatability from DMD mouse models to the clinic remain to be addressed.

## Teaser

**Summary:** This Review discusses central nervous system involvement observed in a subset of individuals with Duchenne muscular dystrophy, highlighting behavioural alterations and brain pathology in humans and mice and evaluating potential therapeutic approaches.

## Introduction

Duchenne muscular dystrophy (DMD) is a severe neuromuscular disorder affecting 1:5000 newborn boys. It is caused by mutations in the X-linked *DMD* gene, which prevent the synthesis of dystrophin. DMD primarily affects boys; female carriers experience milder symptoms. Carriers manifest moderate to severe symptoms in cases of skewed X-inactivation (in which the X-chromosome with the intact *DMD* gene is preferably inactivated), gross chromosomal rearrangements involving translocations between parts of the X-chromosome with the intact *DMD* gene and an autosome, or, rarely, when they also have Turner's syndrome (a chromosomal disorder in which an affected female has a single X-chromosome) (Soltanzadeh et al., 2010; Viggiano et al., 2016). Lack of dystrophin in muscle leads to high susceptibility to damage from early development onwards, resulting in progressive loss of muscle tissue and function (Bradley et al., 1972; Hughes et al., 2019). Consequently, individuals become wheelchair dependent in their teens and die prematurely owing to cardiorespiratory failure around the age of 30 years (Broomfield et al., 2021) (reviewed in Guiraud et al., 2015; Duan et al., 2021). In addition to muscle pathology, ∼30% of individuals with DMD experience cognitive and behavioural deficits that are caused by the absence of brain-specific dystrophin isoforms.

**Fig. 1.**
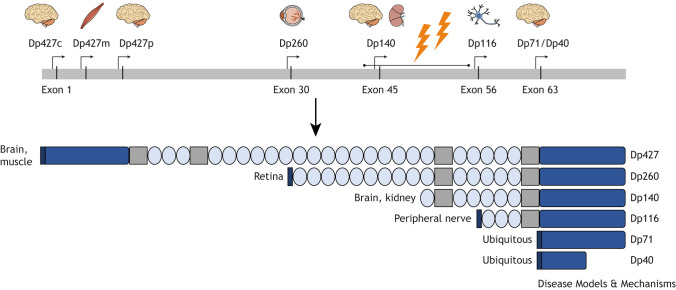
**The human *DMD* gene and the different dystrophin isoforms.** The *DMD* gene contains seven promotors (indicated by the arrows), which give rise to different dystrophin proteins expressed in diverse tissues (indicated by the pictograms above the arrows). Dp427c, Dp427p, Dp140, Dp71 and Dp40 are expressed in the brain. There is one deletion hotspot, indicated by the lightning icons (exon 42-55). Only Dp427 has an N-terminal actin-binding domain (left dark-blue block). Each isoform consists of multiple spectrin-like repeats (light-blue ovals), proline-rich hinge domains (grey blocks) and a C-terminal domain. The C-terminal domain of Dp40 is truncated. Dp427c, Dp427m, Dp427p, Dp260, Dp116, Dp71 and Dp40 have a unique first protein-encoding exon (black block). The unique first exon of Dp140 is not translated because the translation start site is located in exon 51.

The *DMD* gene is the largest gene in the human genome. It consists of 2.4 million base pairs and contains 79 exons (Bello and Pegoraro, 2019). Multiple promotors, spread throughout the gene, give rise to several dystrophin isoforms with distinct functions, sizes and sites of expression (Fig. 1). There are three full-length isoforms of dystrophin, which are expressed in skeletal, cardiac and smooth muscle (Dp427m), cerebral cortex (see Glossary, Box 1; Dp427c) and Purkinje cells (Box 1; Dp427p) (‘Dp427’ indicates dystrophin protein with a size of 427 kDa) (Perronnet and Vaillend, 2010; Waite et al., 2012). Several promotors, located further downstream of the gene, give rise to shorter dystrophin isoforms. Of these, Dp140, Dp71 and Dp40 are expressed in the brain (Lidov et al., 1995; Paúl-González et al., 2021), while Dp260 and Dp116 are expressed in the retina and peripheral nerves, respectively (Rodius et al., 1997; Imamura et al., 2000). In the past decade, knowledge about the complex expression patterns of dystrophin in the brain has significantly expanded (Box 2).

Box 1. Glossary **13-mer/15-mer:** an oligonucleotide that is 13 or 15 nucleotides long.

**Allen Brain Atlas:** high-resolution public database of brain gene expression maps, integrating anatomical and transcriptomic data across development.

**Antisense oligonucleotides (AONs):** synthetic DNA or RNA that binds to RNA to modify gene expression, e.g. by inducing exon skipping.

**AQP4:** a water channel in astrocytes in the brain. It is part of the blood–brain barrier and is important for water balance in the central nervous system.

**BrainSpan Atlas:** database providing gene expression data of the developing human brain across brain regions and ages.

**Cerebello-thalamo-cortical connectivity:** neuronal network linking the cerebellum, thalamus and cerebral cortex, essential for motor and sensory integration.

**Cerebral cortex:** outmost layer of the brain, involved in perception, decision making and memory.

**Default mode network:** network of brain regions that is active during rest and in internal thought processes, such as daydreaming or memory recall.

**Docked vesicular glutamate:** glutamate-filled vesicles located at the presynaptic membrane, ready for neurotransmitter release, essential for excitatory signalling and synaptic transmission.

**GFAP:** an astrocytic intermediate filament protein, used as a marker to assess astrocytic activation.

**Intra-cisterna magna injection:** method to deliver substances directly into the cerebrospinal fluid at the brain's base.

**N-ethyl-N-nitrosourea (ENU)-induced point mutation:** DNA mutation from chemical exposure, causing a single base change.

**Parietal plate:** embryonic brain region that develops into the parietal lobe.

**Purkinje cells:** inhibitory neurons in the cerebellum, essential for motor coordination.

**Revertant muscle fibres:** fibres that express dystrophin despite a genetic mutation, often caused by spontaneous exon skipping.

**ST-elevation myocardial infarction:** heart attack caused by total coronary artery blockage.

**Working memory:** temporary information storage system for reasoning and learning, linked to the prefrontal cortex.

**ZO-1:** tight junction protein crucial for the blood–brain barrier.

Box 2. Dystrophin isoform expression in the human brain Dystrophin expression in the human brain varies based on isoform, brain region and developmental stage. Analysis using the Allen Brain Atlas (Box 1) has shown that dystrophin is highly expressed in the adult human amygdala and hippocampus, with lower levels found throughout the cortex, but particularly in the temporal and frontal lobes (Doorenweerd et al., 2017c). Dystrophin expression in the human pons and cerebellum is low. These observations represent the sum of all dystrophin isoforms, as primers used in this analysis capture the distal part of the *DMD* gene, making distinctions of the individual isoforms impossible.

Expression profiles of individual dystrophin isoforms throughout human development, ranging from 8 weeks post conception to 40 years of age, were studied using the BrainSpan Atlas (Box 1) (Doorenweerd et al., 2017c). Expression of Dp427c and Dp427m is low before birth, slightly increases at ∼2 years of age and then remains low throughout adulthood. In contrast to the murine brain (Górecki et al., 1992; Kueh et al., 2008; Snow et al., 2014), Dp427p is virtually absent in the human brain. Notably, the expression of Dp140 is high in the early foetal brain, but levels drop from late foetal stages onwards. Dp71 and Dp40 are ubiquitously expressed throughout the human brain at high levels during the foetal stages and remain expressed at high levels until adulthood. Catapano et al. (2025) expanded on these findings by mapping dystrophin isoform expression across brain development. They revealed co-expression of multiple dystrophin isoforms within different types of single neurons. For a review of dystrophin and its interactors in the brain, see Tetorou et al. (2024)*.*

In individuals with DMD, the number of dystrophin isoforms they lack depends on the position of the mutation in the *DMD* gene. Notably, mutations in this gene are clustered, with 75% of the deletions occurring between exons 42 and 55. Mutations at the proximal end of the gene (exon 1-43) lead to the absence of Dp427m, Dp427c, Dp427p and Dp260. Distal mutations (intron 44 to exon 79) affect the expression of the full-length and one, multiple or all of the shorter isoforms of dystrophin. Based on the mutation frequency data (Bladen et al., 2015), all individuals with DMD lack the full-length isoforms, ∼40-50% additionally lack Dp140, and up to 10% of individuals lack all dystrophin isoforms (Desguerre et al., 2009; Taylor et al., 2010; Pane et al., 2012; Rasic et al., 2014; Ricotti et al., 2016).

Since the discovery of the *DMD* gene in the 1980s (Hoffman et al., 1987b), its connection to muscle pathology has been studied in great depth. However, research into DMD effects on the human brain has only gained attention in the past decade. Here, we review brain pathology in DMD, focusing mostly on the structural and functional changes in the DMD brain as reported from imaging studies. Next, we summarize information on the DMD animal models that have been used to unravel disease mechanisms, providing an extensive overview of behavioural alterations in domains such as emotional reactivity, learning and memory and social interaction, and the brain pathology in these models. Lastly, we provide an overview of the preclinical developments for treatment of the brain and discuss the limitations and outstanding questions that remain to be addressed.

## Behavioural deficits and brain pathology in DMD

The first description of DMD, dating back to the 1860s, acknowledged the occurrence of brain deficits in a subset of individuals with DMD (Duchenne, 1868). Approximately 30% of them have a higher risk of cognitive impairment, learning disabilities and delayed developmental milestones (Ricotti et al., 2016; Colombo et al., 2017; Darmahkasih et al., 2020). The average IQ of the DMD population (mean=84.76) is one standard deviation below that of the general population (mean=100) (Hinton et al., 2000; Cotton et al., 2001; Cyrulnik et al., 2008; Connolly et al., 2013; Weerkamp et al., 2022). Learning and behavioural conditions occur in individuals with DMD, both with cognitive impairments and with a normal- to high-range IQ (Battini et al., 2018; Fee et al., 2018). Among the cognitive impairments reported in individuals with DMD are difficulties with information processing (Hellebrekers et al., 2020), (verbal) working memory (Box 1) (Hinton et al., 2001; Thangarajh et al., 2020), reading (Hendriksen and Vles, 2006; Lorusso et al., 2013; Astrea et al., 2015) and arithmetic skills (Hinton et al., 2004; Fee et al., 2018). In addition, a subset of individuals with DMD are reported to have comorbidities such as autism spectrum disorder (ASD; ∼6%) (Hendriksen and Vles, 2008; Ricotti et al., 2016; Pascual-Morena et al., 2023), obsessive-compulsive disorder (OCD; ∼7%) (Hendriksen and Vles, 2008; Darmahkasih et al., 2020; Pascual-Morena et al., 2023), attention deficit hyperactivity disorder (ADHD; ∼16%) (Hendriksen and Vles, 2008; Ricotti et al., 2016; Darmahkasih et al., 2020; Pascual-Morena et al., 2023) and a higher chance of experiencing epileptic seizures (5%) (Hoogland et al., 2019; Pascual-Morena et al., 2023).

Increasing evidence suggests that the number of affected dystrophin isoforms correlates with disease severity, with individuals lacking all isoforms being the most severely affected (Taylor et al., 2010; Chamova et al., 2013; Ricotti et al., 2016). Notably, mutations occurring downstream of exon 63 in the *DMD* gene have also been associated with more rapid progression of motor impairment in individuals with DMD, indicating that the specific mutation location influences the severity and timing of functional decline (Chesshyre et al., 2022; Muntoni et al., 2023). However, there remains notable variability in both motor and central nervous system (CNS) outcomes, even among individuals with mutations affecting the 3′ end of the gene, implying that mutation location alone does not fully explain the phenotypic differences (Fang et al., 2025). Additionally, cognitive and behavioural deficits show considerable variability among individuals with DMD; however, these patterns are evident primarily at the group level rather than in individual cases. The DMD-related neuropsychological and neurobehavioral impairments are reviewed in Snow et al. (2013).

### Anatomical and functional consequences of the lack of dystrophin in the human brain

The effect of DMD on human brain morphology and function is not well understood. The sparse and inconsistent post-mortem data that are available for DMD-affected human brains were acquired decades ago and include no reports on gross structural abnormalities. Some DMD brains had increased cortical thickness (Rosman and Kakulas, 1966), neuronal and Purkinje cell loss (Rosman and Kakulas, 1966; Dubowitz and Crome, 1969; Jagadha and Becker, 1988), mononuclear perivascular cuffing (Jagadha and Becker, 1988), cortical and subcortical gliosis (Dubowitz and Crome, 1969; Jagadha and Becker, 1988), reduced dendritic length and branching (Jagadha and Becker, 1988), or cerebral heterotopia (Jagadha and Becker, 1988). More recent data are non-existent owing to the worldwide unavailability of DMD brain tissues.

Neuroimaging techniques [e.g. magnetic resonance imaging (MRI) and positron emission tomography (PET)] have enabled structural, physiological and connectivity analyses in living brains. Individuals with DMD show a decrease in total brain volume and global reductions in grey matter volume, most prominently in the left primary sensorimotor cortex, relative to age- and sex-matched controls (Yoshioka et al., 1980; Al-Qudah et al., 1990; Lv et al., 2011; Doorenweerd et al., 2014). These structural alterations suggest disrupted neurodevelopment, as grey matter plays a central role in both motor and cognitive functions. Although white matter volume seems to be unaffected in DMD, widespread white matter microstructural deficits have been observed using diffusion-weighted imaging. These studies on the structural connectivity of the white matter fibres via water diffusion found increased overall and radial diffusivity in individuals with DMD, paired with a lower fractional anisotropy (Doorenweerd et al., 2014; Preethish-Kumar et al., 2020; Biagi et al., 2021). These changes suggest reduced fibre density, increased membrane permeability and/or decreased structural organization in the brain tissue of individuals with DMD.

In addition to structural abnormalities, functional abnormalities have also been described in individuals with DMD. Resting-state functional MRI shows alterations in default mode network (Box 1) functional connectivity, resulting in hyperconnectivity spread throughout the whole network. This widespread hyperconnectivity may reflect inefficient neural signalling and impaired network segregation. Such patterns mirror those observed in neurodevelopmental conditions such as ADHD, suggesting overlapping mechanisms of altered brain network dynamics (Doorenweerd et al., 2021). Proton spectra studies have reported conflicting data, with both increased and decreased choline compounds observed in different brain regions (cerebellum, hippocampus, and frontal and temporoparietal regions) (Rae et al., 1998; Kreis et al., 2011; Doorenweerd et al., 2017b). As choline influences the synthesis of the neurotransmitter acetylcholine (Prado et al., 2017), changes in its concentrations impact brain network activity (reviewed in Colangelo et al., 2019). Alterations in choline could also play a role in the overall reduction in network efficiency (i.e. reduced speed of information transfer) reported in DMD (Preethish-Kumar et al., 2022). Furthermore, resting-state functional MRI studies have revealed decreased local synchronization of neurons in the motor-related brain areas during spontaneous firing (Lv et al., 2011), reductions in glucose metabolism in the sensorimotor cortex were shown by PET scans (Bresolin et al., 1994; Lee et al., 2002), and decreased excitability of the motor cortex has been shown by the use of transcranial magnetic stimulation (Di Lazzaro et al., 1998). These findings suggest impaired functionality of the motor cortex in individuals with DMD. Lastly, studies on cerebral perfusion have demonstrated reduced cerebral blood flow in individuals with DMD, independent of age or grey matter volume reduction (Doorenweerd et al., 2017a). Perfusion levels were lower in individuals also lacking the Dp140 isoform compared to those in individuals only lacking Dp427. Whereas some studies reported that the reduced grey matter volume and altered structural connectivity are more pronounced in individuals with DMD who lack both Dp427 and Dp140 (Doorenweerd et al., 2014; Preethish-Kumar et al., 2022), others have either failed to confirm these findings or had a low sample size (Lv et al., 2011; Biagi et al., 2021; Govaarts et al., 2024). In addition, no clear correlation between the neuroimaging outcomes and the severity of cognitive and behavioural defects has been found. It should, however, be noted that the vast majority of individuals with DMD chronically use corticosteroids, which are known to negatively affect behaviour (Angelini, 2007; Counterman et al., 2022). Recent studies on chronic corticosteroid use have revealed correlations between corticosteroid dose and regimen and the extent of alterations in brain volume and white matter microstructure (Van Der Meulen et al., 2022; Geuens et al., 2023).

In summary, neuroimaging studies have consistently revealed structural and functional differences between the brains of individuals with DMD and sex- and age-matched controls. However, these insights remain largely correlative, limiting our ability to draw firm conclusions about causality or the underlying mechanisms driving clinical symptoms. This gap could be attributed to several methodological challenges, including the small sample sizes of most studies (often ∼15 participants), which restrict statistical power and the ability to stratify by mutation subtypes and/or corticosteroid use. Variability in steroid regimens, dosages and duration further complicates interpretation. These limitations underscore the need for larger, longitudinal and methodologically harmonized imaging studies to better clarify the relationship between brain involvement and clinical presentation of cognitive and behavioural deficits in DMD. In this context, DMD animal models offer valuable opportunities to explore brain isoform-specific dystrophin functions, providing complementary insights that may help bridge knowledge gaps and inform human studies.

## Animal models of DMD

Invaluable insights into the consequences of a lack of dystrophin in the brain have been obtained through preclinical studies in a variety of dystrophic animal models. The majority of DMD research has been conducted on mouse models, as mice are fast breeders, easy to maintain in large numbers and well characterized on a behavioural level. The *Dmd* gene is well conserved in mice (Hoffman et al., 1987a). However, less is known about the extent to which the spatiotemporal expression patterns of the dystrophin isoforms are preserved between man and mice. Furthermore, the murine brain is less complex than the human brain, leading to differences in cortical organization and connectivity (Wong et al., 2023).

Larger-animal models have also been used in the DMD field, primarily to investigate the muscle pathology. In particular, the porcine, canine and rat models have also been used to investigate the DMD brain. The porcine model offers high translational relevance owing to anatomical and physiological similarities to humans. In the DMDΔ52 porcine model (carrying a deletion of exon 52 leading to a lack of Dp427 and Dp140 in the brain), reduced exploration and possibly a small delay in learning a new task have been reported (Stirm et al., 2021). It is unclear whether other types of behaviour are affected.

There are multiple DMD canine models, which all closely capture the muscular aspects of the disease. Studies on the canine brain are limited to the DE50-MD dog (which has a missense mutation in the donor splice site of exon 50 and consequently lacks only Dp427), which have reported reduced attention, problem solving and exploration of novel objects (Crawford et al., 2022), as well as regional reductions in grey matter and increased ventricular volume (Crawford et al., 2023). Investigations on these larger-animal models are constrained by their high costs and ethical concerns.

The use of DMD rat models has grown over the past years, as they offer a cheaper and easier to maintain alternative. DMD rats have a severe muscular dystrophy that more closely mimics that of individuals with DMD than the mildly affected DMD mouse models. Furthermore, the rat models also have a higher translational value owing to their larger brain size and more complex behaviour. Behavioural impairments have been studied in the *Dmd^mdx^* rat (carrying an out-of-frame mutation in exon 23 of the *Dmd* gene, and lacking Dp427), which consist of altered fear response (Caudal et al., 2020), and neurochemical and local changes in brain structures (Xu et al., 2020). Although a rat model lacking Dp140 has been previous developed (R-DMDdel52 rat, which carries a 188 bp deletion with premature stop codon in exon 52 in the rat *Dmd* gene), analyses have so far solely focused on its muscular pathology.

Because most of our knowledge on the DMD brain has been obtained from mouse models, we will focus on them in this Review.

### Dystrophin-deficient mouse models

#### Mouse models lacking Dp427

The C57BL/10ScSn-*Dmd^mdx^*/J (*mdx/*bl10) mouse was the first DMD model in which cognitive abnormalities were reported (Muntoni et al., 1991), and it has since been widely used in DMD research. The *mdx*/bl10 mouse has a point mutation in exon 23 of the *Dmd* gene and consequently lacks Dp427 (Sicinski et al., 1989). *Mdx*/bl10 mice exhibit skeletal muscle pathology, including necrosis, inflammation and central nucleation, with relatively mild functional impairment owing to efficient regeneration and compensatory mechanisms. Cardiac and smooth muscle are also affected. Although this pathology could potentially influence behaviour in terms of reduced exploration and/or activity (Vaillend et al., 1995, 2004), it is rather mild compared to that seen in human DMD. *Mdx*/bl6 mice, generated on the C57BL/6J genetic background, carry the same mutation as the *mdx*/bl10 model and exhibit a similar degree of muscle pathology*.*

B6Ros.Cg-*Dmd^mdx-5Cv^*/J (*mdx^5cv^*) mice have an N-ethyl-N-nitrosourea (ENU)-induced point mutation (Box 1) in exon 10 and consequently lack Dp427 (Im et al., 1996). In contrast to the *mdx*/bl10 and *mdx*/bl6 models, their muscle pathology and functioning are slightly more impaired, likely owing to the absence of revertant muscle fibres (Box 1) (Danko et al., 1992; Beastrom et al., 2011).

The genetic background of animals is an important factor to consider when assessing their behaviour, which is also true in the context of DMD (Wolfer and Lipp, 2000; Jacobson and Cryan, 2007; Seemiller et al., 2021). Differences between distinct C57BL strains have been reported in expression profiles (Flynn et al., 2021; Mortazavi et al., 2022), sensitivity to seizure induction (McLin and Steward, 2006) and the brain glutamatergic system (Deacon et al., 2007). This probably adds to the inconsistencies reported between *mdx/*bl10, *mdx*/bl6 and *mdx^5cv^* mice, discussed in this Review.

#### Mouse models lacking Dp427 and Dp140

The consequences of the lack of Dp427 and Dp140 have been primarily studied in the *mdx52* mouse, which has a deletion of exon 52 (Araki et al., 1997) and the B6Ros.Cg-*Dmd^mdx-4Cv^*/J (*mdx^4cv^*) mouse, which carries a nonsense mutation in exon 53 (Im et al., 1996). Although Dp140 is not expressed in muscle, *mdx52* mice display more impaired executive motor function than do *mdx*/bl10 mice (Chesshyre et al., 2022). The cerebellum (a main site of Dp140 expression) and/or the cerebello-thalamo-cortical connectivity (Box 1) are hypothesized to play an important role in this phenomenon, considering the crucial role of the cerebellum in the timing and control of goal-directed movements. In individuals with DMD, similar correlations between the mutation site and motor function have been found in one (Chesshyre et al., 2022), but not in another (Thangarajh et al., 2021), study. This discrepancy could be due to differences in sample size [larger sample size in Chesshyre et al. (2022), in which a correlation was found] or in the tests used to determine muscle function. Owing to the position of the mutation in the *mdx52* mutant, these mice also lack Dp260, which might account for the observed altered visual processing (Barboni et al., 2021). Muscle performance or visual processing have not yet been functionally assessed in *mdx^4cv^* mice.

These DMD mouse models' phenotypes highlight the importance of recognizing CNS involvement in executive motor function in DMD, which influences measurable behaviour during assessments. Executive motor function should, therefore, be taken into consideration when reviewing behaviour in DMD mice, especially those lacking Dp260 and Dp140.

#### Mouse models lacking all dystrophin isoforms or Dp71 only

The *DMD-null* mouse lacks all dystrophin isoforms owing to the deletion of the entire genomic region of the *Dmd* gene (Kudoh et al., 2005). These mice display severe muscle hypertrophy but have functional deficits that are similar to those observed in the *mdx52* model. By contrast, individuals with DMD, who lack all dystrophin isoforms, experience worse functional deficits than do those individuals who lack only isoforms Dp427 and Dp140 (Chesshyre et al., 2022). The underlying cause of this discrepancy is unknown.

The Dp71-null mouse was created by replacing part of the first exon of Dp71 (Kudoh et al., 2005). Although this model does not recapitulate the human condition, as no human *DMD* mutations have been identified that exclusively affect Dp71, it provides insight into the specific functions of Dp71. Dp71-null mice have healthy muscles owing to unaffected expression of Dp427m (Helleringer et al., 2018), but exhibit altered retinal functioning (Barboni et al., 2020). This deficit has not yet been studied in *DMD-null* mice.

### Behavioural deficits in DMD mouse models

In addition to motor impairment, a subset of individuals with DMD experiences behavioural and cognitive difficulties to varying degrees. Although impairments in academics such as mathematics and reading cannot be studied in animal models, many of the behavioural domains affected in individuals with DMD have been investigated in DMD mice. In the following section, we review the behavioural phenotypes observed in DMD mouse models, with a focus on emotional reactivity, learning and memory, and social behaviour. A brief overview of all behavioural deficits observed in different DMD mouse models can be found in Table 1, while Box 3 offers explanations of the behavioural assays discussed.

Box 3. Behavioural assays **Emotional reactivity**

**Dark–light box** is an anxiety test based on the time spent in illuminated versus dark compartments of a box. Often ignores behaviour in the dark zone.

**Elevated plus maze** measures anxiety via the time spent in open versus closed arms of an elevated maze (plus shape). The test is highly dependent on motor function.

**Foot shock** measures the freezing response (unconditional) after applying a mild shock to the feet of the mice. Longer freezing time indicates a more severe stress response. This test causes significant stress to the animals, and significant variations are possible owing to individual pain sensitivity in animals.

**Forced swim test** measures despair (often labelled as depression) via immobility in water (without the option to escape). Interpretation of the test is debated.

**Light stimulus** assesses response to a sudden bright light, causing anxiety. The consequences of sensory sensitivity (or retinal abnormalities) and anxiety are hard to separate.

**Open field** measures locomotion and anxiety via centre versus perimeter exploration in a large open arena. Staying closer to the walls and decreased exploration correspond with higher anxiety levels. It is hard to separate low activity caused by anxiety and motor impairments.

**PhenoTyper cages** are automated home cages for long-term behaviour testing. The interpretation can be complex owing to the vastness of the data.

**Restrained unconditioned fear response** measures the fear response after physical restraint (e.g. being held upside down) without any interplay of learning or associated behaviour. Longer freezing time indicates a more severe stress response. This test is considered to be stressful for DMD mice.

**Tail suspension test** measures despair (often labelled as depression) via immobility when hanging for a prolonged time by the tail. Causes high stress, and interpretation is debated.

**Learning and memory**

**Auditory fear conditioning** associates a tone with an adverse stimulus (e.g. foot shock) and measures freezing upon re-exposure to the tone without the stimulus. This test is dependent on intact hearing and can cause stress induced by the shock and the auditory cue.

**Bar pressing task** is an operant conditioning task that requires mice to press a lever for rewards, to test motivation and learning. Learning period can be time consuming and is highly based on activity and motivation.

**Barnes maze** assesses spatial learning and memory using a platform with multiple escape holes, one of which leads to a hidden escape box. Relocation of the escape hole allows for assessment of learning flexibility. This test is less stressful than the Morris water maze, but more susceptible to the effect of motivation.

**Cognition wall** is an operant task used to assess learning, learning flexibility, memory or attention in an automated manner. This test requires an advanced setup and long training periods.

**Contextual fear** tests memory by associating an environment with an adverse stimulus (e.g. foot shock), measuring freezing on re-exposure of the context without the stimulus.

**Foot shock** measures learning and fear memory by conditioning mice to avoid an area associated with a foot shock, allowing testing of learning in a high-stress environment.

**Light avoidance** measures avoidance learning by pairing a specific shelter entrance with a bright light. Mice learn to shift their entry to avoid the stimulus. This test is affected by initial preference and general activity.

**Morris water maze** tests spatial learning and memory in a pool with a hidden platform. Learning flexibility can be assessed by repositioning the platform. This test requires high motor function use.

**Nose poke avoidance** measures learning and impulse control using operant conditioning; mice learn to avoid poking a lit port. This test requires extensive training, and motor impairments can affect performance.

**Nose poke task** is similar to bar pressing but uses nose pokes instead.

**Novel object recognition** measures memory by assessing time spent exploring new versus familiar objects (or object locations). This test can be influenced by initial preference for novelty or reduced exploration due to anxiety or motor deficits.

**Radial maze** is used to assess spatial working memory and to reference memory by measuring the selection of maze arms for rewards. Performance can be influenced by motivation levels and motor deficits.

**T-maze test** is used for spatial learning and working memory via exploration of arms in a T-shape. This test can be done by either forced or spontaneous alternation and depends on motivation.

**X-maze test** is similar to the T-maze test, but in an X-shape. This test is dependent on motivation.

**Social interaction**

**Pup separation** measures vocalization or stress behaviour after separating a pup from its mother. This test induces stress to pups.

**Social defeat** tests stress resilience and behaviour after repeated exposure to an aggressive animal. Stress can vary based on the behaviour of the aggressor. Owing to low cardiac resilience of DMD mice, this test can lead to fatalities.

**Three-chamber social interaction** assesses sociability and social novelty preferences by offering choices in a controlled social setting. Behaviour can be affected by locomotor activity or sensory deficits.

**Table 1.** Behavioural deficits in mouse models of Duchene muscular dystrophy

Behaviour			Lack of Dp427			Lack of Dp427+Dp140*		Lack of all isoforms	Lack of Dp71	
Domain		Type of test	*mdx*/bl10	*mdx*/bl6	*mdx^5cv^*	*mdx^4cv^*	*mdx52*	*DMD-null*	Dp71-null	References
**Pathology**	Muscle function		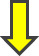	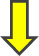	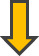	ND	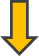	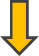	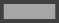	Danko et al., 1992; Vaillend et al., 1995, 2004; Beastrom et al., 2011; Helleringer et al., 2018; Chesshyre et al., 2022
**Motional reactivity**	Fear	Restrained, shock	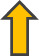	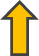	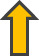	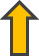	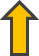	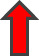	ND	Sekiguchi et al., 2009; Razzoli et al., 2020; Zhang et al, 2020; Hashimoto et al., 2022; Verhaeg et al., 2025
	Anxiety	DL/OF/EPM/light stimulus	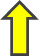	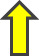	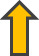	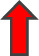	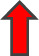	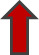	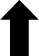	Vaillend et al., 1995; Daoud et al., 2009; Sekiguchi et al., 2009; Manning et al., 2014; Remmelink et al., 2016; Vaillend and Chaussenot, 2017; Comim et al., 2019; Saoudi et al., 2021; Verhaeg et al., 2025
	Depressive behaviour	FST/TST	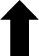 #	ND	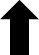	ND	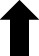	ND	ND	Kudoh et al., 2005; Vaillend and Chaussenot, 2017; Helleringer et al., 2018; Comim et al., 2019
	Spontaneous behaviour	PhenoTyper cages	ND	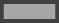	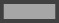	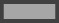	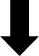 #	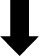	ND	Verhaeg et al., 2025
**Working memory**	Working memory	X-maze, T-maze	ND	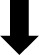	ND	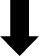	ND	ND	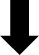	Chaussenot et al., 2019; Verhaeg et al., 2024
**Passive avoidance**	Passive avoidance	Foot shock	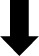	ND	ND	ND	ND	ND	ND	Remmelink et al., 2016; Lewon et al., 2017; Vaillend and Chaussenot, 2017
**Learning**	Spatial learning	MWM, BM	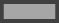	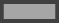	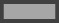	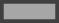	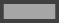	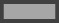	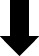	Sesay et al., 1996; Vaillend et al., 2004; Daoud et al., 2009; Remmelink et al., 2016; Bagdatlioglu et al., 2020; Verhaeg et al., 2025
	Fear learning	LA, NPA, CF, ACF	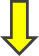	ND	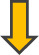	ND	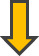	ND	ND	Daoud et al., 2009; Remmelink et al., 2016; Lewon et al., 2017; Vaillend and Chaussenot, 2017; Saoudi et al., 2021
	Food reward learning	CW, BPT, NP, radial maze	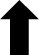	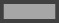	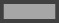	ND	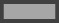	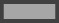	ND	Vaillend et al., 1995, 1998; Vaillend and Ungerer, 1999; Remmelink et al., 2016; Lewon et al., 2017; Dickson and Mittleman, 2019
**Recall**	Short-term recall	T-maze, NOR	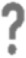	ND	ND	ND	ND	ND	ND	Vaillend et al., 1995, 2004; Bagdatlioglu et al., 2020
	Long-term recall (spatial)	T-maze, MWM, BM	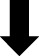	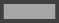	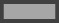	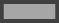	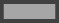	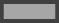	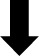	Sesay et al., 1996; Vaillend et al., 2004; Daoud et al., 2009; Remmelink et al., 2016; Chaussenot et al., 2019; Bagdatlioglu et al., 2020; Verhaeg et al., 2024, 2025
	Long-term recall (recognition)	NOR	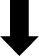	ND	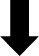 #	ND	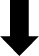 #	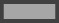	ND	Vaillend et al., 1995, 2004; Comim et al., 2019; Bagdatlioglu et al., 2020
**Cognitive flexibility**	Learning flexibility	BM	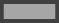	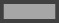	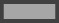	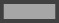	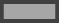	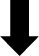	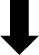	Sesay et al., 1996; Chaussenot et al., 2015, 2019; Remmelink et al., 2016; Engelbeen et al., 2021; Verhaeg et al., 2025
	Food-rewarded flexibility	CW, NP	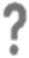	ND	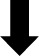	ND	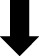	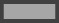	ND	Vaillend et al., 1995; Remmelink et al., 2016; Lewon et al., 2017; Dickson and Mittleman, 2019; Engelbeen et al., 2021; Verhaeg et al., 2025
**Extinction**	Extinction learning	BPT	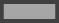	ND	ND	ND	ND	ND	ND	Vaillend and Ungerer, 1999; Vaillend and Chaussenot, 2017; Dickson and Mittleman, 2019
**Social interaction**	Sociability	3C	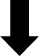	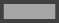	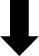	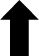	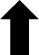	ND	ND	Miranda et al., 2015; Alexander et al., 2016; Hashimoto et al., 2022; Verhaeg et al., 2025
	Social novelty seeking	3C	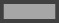	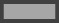	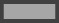	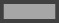	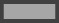	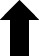	ND	Miranda et al., 2015; Alexander et al., 2016; Hashimoto et al., 2022; Verhaeg et al., 2025
	Social stress response	Social defeat	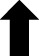	ND	ND	ND	ND	ND	ND	Razzoli et al., 2020
	USV	Pup separation	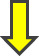	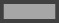	ND	ND	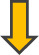	ND	ND	Miranda et al., 2015; Hashimoto et al., 2022

The arrow direction indicates whether behaviour is increased (up) or decreased/impaired (down) compared to that of WT mice. Differences between mouse models are represented by the colours of the arrows, with yellow indicating the least severe deficit and dark red indicating the strongest deficit. Black arrows represent a single result, which does not allow for direct comparisons between models. A question mark indicates conflicting data in the literature. The combination of an arrow and a hash sign indicates a trend. Grey horizontal bars indicate that no differences were found. ‘ND’ indicates that the behaviour has not been investigated in this model.

*Mice also lack Dp260.

3C, three-chamber social interaction task; ACF, auditory cued fear; BM, Barnes maze; BPT, bar pressing task; CF, contextual fear; CW, cognition wall; DL, dark-light choice test; EPM, elevated plus maze; FST, forced swim test; LA, light avoidance; MWM, Morris water maze; NP, nose pokes; NPA, nose poke avoidance; ND, not determined; NOR, novel object recognition task; OF, open field; TST, tail suspension test; USV, ultrasonic vocalization.

#### Emotional reactivity

Various tests have been used to assess emotional reactivity in DMD mouse models (Box 3). The most prominent phenotype of the *mdx*/bl10 mouse is the severe fear response, in which a short stressor (i.e. manual restraint) instantly causes freezing behaviour that lasts for at least 1 h (Sekiguchi et al., 2009; Yamamoto et al., 2010; Vaillend and Chaussenot, 2017; Razzoli et al., 2020; Saoudi et al., 2021). This altered freezing response can be exhibited as early as 36 days after birth (Sekiguchi et al., 2009). Only intensive repetition [>16 times per day (Vaillend and Chaussenot, 2017)] or activation of the territorial drive via strong odours of unfamiliar mice can reduce, but not extinguish, freezing behaviour (Yamamoto et al., 2010). The mechanism underlying the severe fear response is not fully understood. Vaillend and Chaussenot (2017) showed that wild-type (WT) mice exhibit similar physiological stress responses to the restraint itself, as do *mdx*/b10 mice (reflected by comparable acetylcholine levels). This finding indicates that this increased freezing stems from a more downstream reaction to the stress, i.e. it is not caused by the initial stress signal but by abnormal processing (e.g. via fear memory processing, emotion regulation or motor inhibition pathways) of that signal in the brain. *Mdx/*bl6 and *mdx^5cv^* mice, which lack Dp427 (Hashimoto et al., 2022; Verhaeg et al., 2025), and *mdx52* and *mdx^4cv^* mice, which lack Dp140 in addition to Dp427 (Zhang et al., 2020; Saoudi et al., 2021; Hashimoto et al., 2022; Verhaeg et al., 2025), exhibit similar freezing responses to those observed in *mdx*/bl10 mice. Interestingly, this freezing response is further increased in *DMD-null* mice (Verhaeg et al., 2025).

Although the lack of Dp427 appears to significantly affect the unconditioned fear response, it does not seem to affect other types of emotional behaviour as strongly. Studies in DMD mouse models have struggled to conclusively detect anxiety-like behaviour in *mdx*/bl10 mice (Vaillend et al., 1995; Sekiguchi et al., 2009; Comim et al., 2019) and to produce consistent results, detecting anxiety-like behaviour in the open field test, but not in the dark–light box or the elevated plus maze (Manning et al., 2014; Remmelink et al., 2016; Vaillend and Chaussenot, 2017; Saoudi et al., 2021). This highlights the subtle nature of the anxiety phenotype in *mdx*/bl10 mice and suggests that the environment and test protocols both play a crucial role in the consistent detection of this deficit. Lack of Dp140 further deteriorates the anxious behaviour, as both *mdx^4cv^* and *mdx52* mice show stronger anxiety responses than do *mdx*/bl6 and *mdx^5cv^* mice, respectively (Saoudi et al., 2021; Verhaeg et al., 2024, 2025). This anxious behaviour is even further enhanced in *DMD-null* mice (Verhaeg et al., 2025) and, to some extent, in Dp71-null mice (Daoud et al., 2009), indicating worsening of anxiety with the lack of each brain-related dystrophin isoform.

Apart from the fear response and anxiety-like behaviour, depressive-like behaviour has also been studied in *mdx*/bl10 mice. However, changes in despair and in learned helpnessless are inconsistent in this model, as they have been reported (Comim et al., 2019) but could not be replicated (Vaillend and Chaussenot, 2017), possibly owing to high stress reactivity. *Mdx^5cv^* and *mdx52* mice appear to have enhanced behavioural despair but no alterations in learned helplessness (Saoudi et al., 2024). Dp71-null mice show no alterations in behavioural despair (Helleringer et al., 2018), and *DMD-null* mice have yet to be evaluated for depressive-like behaviour.

Recent research also shows that *DMD-null* males exhibit restless behaviour, as well as altered day/night rhythms, movement and rest patterns, which have not been observed in *mdx^5cv^* or *mdx52* mice (Verhaeg et al., 2025). This specific type of behaviour has not been reported in individuals with DMD; however, it is in line with the observation that more distal mutations lead to more severe cognitive and behavioural deficits in both mouse models and humans (Vaillend et al., 2025).

#### Learning and memory

As some individuals with DMD exhibit learning difficulties, learning and memory have been studied in DMD mouse models. Some learning and memory deficits in individuals with DMD, e.g. word memory and conceptual knowledge, cannot be directly tested in mice owing to species-specific limitations. However, other domains, such as working memory, recall and learning flexibility, are more similarly assessed and therefore more easily translatable between mice and humans.

Working memory is impaired in *mdx*/bl6 (Verhaeg et al., 2024)*, mdx^4cv^* (Verhaeg et al., 2024) and Dp71-null (Chaussenot et al., 2019) mice, but it remains unclear whether the severity of this impairment differs between models. Initial learning of a task or location is unaffected in *mdx*/bl10 and *mdx^5cv^* mice (Sesay et al., 1996; Vaillend et al., 2004; Remmelink et al., 2016; Bagdatlioglu et al., 2020). However, memory deficits become apparent when this information needs to be recalled at a later time, with the extent of the effect depending on the time delay. Data on the short-term memory recall are inconclusive for *mdx*/bl10 mice (Vaillend et al., 1995, 2004; Bagdatlioglu et al., 2020) and might be strongly influenced by the context of the test, as differences in delay times and learning protocols could explain why one study reported differences (Bagdatlioglu et al., 2020), whereas another utilizing the same object recognition test did not (Vaillend et al., 2004). Long-term memory recall (>24 h delays) seems to be more robustly affected in these mice, in terms of their spatial memory [hippocampus dependent (Sesay et al., 1996; Vaillend et al., 2004; Remmelink et al., 2016; Bagdatlioglu et al., 2020)] and recognition memory [hippocampus independent (Vaillend et al., 1995, 2004; Comim et al., 2019; Bagdatlioglu et al., 2020)]. Spatial learning and memory are not affected in *mdx52*, *mdx^4cv^* or *DMD-null* mice (Verhaeg et al., 2024, 2025), whereas deficits have been found in Dp71-null mice (Daoud et al., 2009; Chaussenot et al., 2019).

Long-term recall deficits in other types of memory have also been observed in *mdx*/bl10 mice (Muntoni et al., 1991; Coccurello et al., 2002; Vaillend and Chaussenot, 2017; Comim et al., 2019). In contrast to WT mice, *mdx*/bl10 mice fail to show strong reactions in fear learning and in passive avoidance in response to an auditory stimulus. Interestingly, this lack of auditory reactivity in *mdx*/bl10 mice is not observed in response to a visual stimulus, which elicits a similar response to that seen in WT mice (Daoud et al., 2009; Remmelink et al., 2016; Lewon et al., 2017; Vaillend and Chaussenot, 2017; Saoudi et al., 2021). In *mdx^5cv^* and *mdx52* mice, the lack of response to auditory stimuli is even more pronounced (Saoudi et al., 2021). Notably, 5-week-old *mdx*/bl10 mice exhibit partial hearing loss (Raynor and Mulroy, 1997; Chen et al., 2002). As such, the lack of their response to auditory stimuli might be due to the lower impact of the auditory cue instead of a learning deficit. Because *mdx^5cv^* and *mdx52* mice exhibit more severe auditory deficits than do *mdx*/bl10 mice, genetic background might also play a role in either fear learning and/or in auditory processing.

Comparison of negative and positive reinforcement learning in DMD mouse models shows that negative reinforcement is not impacted by loss of dystrophin, whereas positive reinforcement, such as a food reward, can lead to improved performance in *mdx*/bl10 compared to WT mice (Vaillend et al., 1998; Lewon et al., 2017; Dickson and Mittleman, 2019). However, some studies have failed to replicate this increased performance in *mdx*/bl10 mice (Vaillend et al., 1995; Vaillend and Ungerer, 1999; Remmelink et al., 2016). This lack of replication could be explained by the results of Lewon et al. (2017), who have shown that the increased performance of *mdx*/bl10 mice in this task only occurs when mice are food deprived before the start of the task (Lewon et al., 2017). *Mdx*/bl10 mice might be more motivated to collect food owing to their increased metabolic rate, caused by the continuous need to repair their muscle tissue (Radley-Crabb et al., 2011, 2014; Stapleton et al., 2014). They consequently perform better after food deprivation. Because the nature of the stimulus and the motivational drive both play an important role in learning motivation tasks, it can be hard to draw conclusions on learning capabilities in tasks that involve positive or negative stimuli.

Cognitive flexibility, the ability to learn new information that is in conflict with earlier acquired information, does not seem to be impaired in DMD models that lack Dp427 or both Dp427 and Dp140 (Sesay et al., 1996; Chaussenot et al., 2015; Engelbeen et al., 2021). However, *mdx*/bl10 mice seem to retain old information better than do WT mice and rely on this when newly learned behaviour does not yield the desired result in the Barnes maze (Remmelink et al., 2016). *DMD-null* and Dp71-null mice show a delay in cognitive flexibility (Chaussenot et al., 2019; Verhaeg et al., 2025). The introduction of positive food reinforcement in cognitive flexibility tasks has led to contradictory results, in which *mdx*/bl10 mice exhibit increased performance (Lewon et al., 2017), decreased performance (Vaillend et al., 1995; Remmelink et al., 2016) or no differences relative to WT mice during reversal tasks (Dickson and Mittleman, 2019; Engelbeen et al., 2021). These findings suggest altered motivation or affected reward processing in the absence of dystrophin, which is also relevant in individuals with DMD, who are reported to sometimes struggle with adapting to changing rules or expectations (Donders and Taneja, 2009; Ricotti et al., 2016).

Extinction learning, the unlearning of earlier acquired information in the absence of new options, does not seem to be affected in *mdx*/bl10 mice (Vaillend and Ungerer, 1999; Dickson and Mittleman, 2019), nor does taste aversion learning (Vaillend and Chaussenot, 2017). In individuals with DMD, difficulties in flexibility may underlie challenges in adapting to new routines, learning from feedback or eliminating unwanted behaviours (Donders and Taneja, 2009; Ricotti et al., 2016).

#### Social interaction

Mouse models of DMD have been observed in interaction experiments in different social contexts to investigate ASD, as a comorbidity of DMD. Studies have found that 8-week-old, but not 5-month-old, *mdx*/bl10 mice exhibit no preference for social interaction, in contrast to WT mice, which show a preference for social interaction at both ages (Stapleton et al., 2014; Alexander et al., 2016). The social interaction behaviour of *mdx*/bl10 mice is strongly influenced by the sex and genotype of the interacting mouse, as *mdx*/bl10 mice exhibit abnormal behaviour in direct interactions with male and female *mdx* mice, but not with male WT mice. This response is suggestive of a submissive response in *mdx*/bl10 mice, which is more easily influenced than that in WT controls. As in *mdx*/bl10 mice, *mdx^5cv^* mice show a similar lack of preference for social interaction at 5 weeks of age (Alexander et al., 2016). Interestingly, *mdx52* mice show increased tendencies towards sociability compared to WT and *mdx*/bl10 mice (Hashimoto et al., 2022), possibly owing to the abnormal presynaptic glutamatergic transmission modulated by the lack of Dp140 in the basolateral amygdala, as stated by the authors.

*Mdx*/bl10 mice seem specifically vulnerable to social stress, which is in line with reports from individuals with DMD, who show severe stress, anxiety and social problems (Hinton et al., 2006). When confronted with the odour of an intruder in their home cage, they show signs of stress, as indicated by a freezing response (Miranda et al., 2015). When experiencing social defeat followed by prolonged sensory housing (meaning that animals can still see, hear and smell each other, but are prohibited from direct contact by a transparent wall), *mdx*/bl10 mice develop heart damage and other cardiovascular responses, resulting in death within 2 days (Razzoli et al., 2020), highlighting an association between stress vulnerability and cardiomyopathy. Although this association has not yet been made in individuals with DMD, it has been in other situations, including individuals with ST-elevation myocardial infarction (Box 1) (Reinstadler et al., 2016). Hypotension, as a result of shock, is associated with conditions such as stroke, cardiac arrest and respiratory failure, which are among the most common causes of death in DMD (Winterholler et al., 2016; Cheeran et al., 2017). DMD could, therefore, be associated with failure to mediate proper autonomic/cardiovascular responses, leading to extensive physical consequences in response to stress.

Lastly, alterations in ultrasonic calls, which are similar to those seen in ASD mouse models, have been found in *mdx*/bl10, but not *mdx*/bl6, pups and adult mice (Miranda et al., 2015; Hashimoto et al., 2022). In *mdx52* mice, these alterations are even more apparent (Hashimoto et al., 2022), underscoring the already established (although indirect) link between DMD and ASD in mice and humans.

### Neuroanatomical and physiological consequences of a lack of dystrophin in the brain

As discussed earlier, neuroimaging techniques have revealed a range of structural and functional abnormalities in the brains of individuals with DMD. Building on these human findings, neuroimaging studies in DMD mouse models, particularly the *mdx*/bl10 mouse, have explored the similarities in alterations between the murine and human DMD brain. Via T1 and T2 neuroimaging, structural changes in the brains of young adult *mdx*/bl10 and *mdx52* mice have been investigated. Little-to-no abnormalities were observed in the volume of the whole brains or individual brain regions of these mice (Bagdatlioglu et al., 2020; Saoudi et al., 2021). However, from 12 months of age onwards, total brain volume was reported to increase in *mdx*/bl10 mice (Dunn and Zaim-Wadghiri, 1999; Miranda et al., 2009; Bagdatlioglu et al., 2020), accompanied by increases in certain cortical structures, such as the basolateral amygdala and the ventricles (Miranda et al., 2009; Bagdatlioglu et al., 2020). Contrastingly, total brain volume in individuals with DMD is decreased (Doorenweerd et al., 2014). The underlying cause of this discrepancy is unknown. Twelve-month-old *mdx*/bl10 mice were also reported to have a rounder head [as also seen in individuals with DMD (Straathof et al., 2014)], as well as a shorter nasal plate and wider parietal plate (Box 1).

Neuroimaging techniques have also been used to assess structural connectivity in the brains of DMD mouse models via diffusion tensor imaging. This technique determines the white matter fibres via water diffusion. Medial diffusion, indicating how freely water can move around in the brain, is increased in individuals with DMD, suggesting reduced fibre density, increased membrane permeability and/or decreased structural organization (Doorenweerd et al., 2014; Preethish-Kumar et al., 2020; Biagi et al., 2021). By contrast, medial diffusion is decreased in *mdx/*bl10 mice, indicating increased fibre density and structural organization, or decreases in membrane permeability (Goodnough et al., 2014). This difference might be explained by the fact that, in individuals with DMD, only the white matter tracts were analysed, whereas in mice the whole brain was measured. Moreover, regional increases in medial diffusion have been reported in the cortex of *mdx*/bl10 mice, along with decreases in fractional anisotropy in the hippocampus (Xu et al., 2015), suggesting high heterogeneity in water diffusion in the brain of *mdx*/bl10 mice.

To our knowledge, resting-state functional MRI, to investigate functional connectivity, has not been performed in DMD mouse models. However, multiple studies have been conducted on the choline/acetylcholine network in the brain of DMD mouse models by assessing choline either through *in vivo* spectroscopy or via postmortem biochemical assays. Increased choline compounds, mostly restricted to the cerebellum and hippocampus, have been reported in older (>6 months) *mdx*/bl10 mice (Rae et al., 2002). Reductions in the enzyme acetylcholinesterase (which is responsible for breaking down acetylcholine) have also been reported in the cortex of *mdx* mice (Tracey et al., 1996; Comim et al., 2011). This reduction suggests a potential disruption in cholinergic neurotransmission, likely contributing to an imbalance in the choline–acetylcholine cycle. These findings imply that dystrophin deficiency can lead to altered cholinergic signalling, particularly in brain regions involved in motor coordination, memory and higher-order cognitive functions.

Alterations in glucose metabolism have been described in the brains of individuals with DMD, and in those of *mdx/*bl10 mice, resulting in increased cellular flux of ^13^C via oxidative glucose metabolism (Rae et al., 2002). This suggests a shift in neuronal energy demands or mitochondrial function. Furthermore, *mdx*/bl10, *mdx52* and Dp71-null mice show reductions in docked vesicular glutamate (Box 1) (Daoud et al., 2009; Hashimoto et al., 2022), leading to enhanced glutamatergic transmission and altered synaptic responses. This synaptic imbalance may contribute to neuronal hyperexcitability, network instability and excitotoxic stress, which are known DMD pathologies. Together, these metabolic and synaptic changes indicate that dystrophin deficiency disrupts both energy homeostasis and neurotransmitter regulation, potentially underlying the cognitive and behavioural impairments observed in some individuals with DMD.

Electrophysiological studies have also been conducted to investigate neuronal transmission in the brains of DMD mouse models. These studies have shown that, in *mdx*/bl10 mice, the excitability of Purkinje cells and the range of their synaptic transmission are reduced (Kreko-Pierce and Pugh, 2022). Many studies have focused on the role of GABA in excitatory/inhibitory signalling balance in the *mdx/*bl10 brain, i.e. the balance between excitatory neurotransmission (primarily mediated by glutamate) and inhibitory neurotransmission (primarily mediated by GABA). In healthy brains, this balance is crucial for the coordination and timing of neuronal firing, which is essential for many brain processes including motor control and cognition. Dystrophin plays a crucial role in this process by serving as a scaffold that anchors GABA receptor subunits at the synaptic membrane, thereby stabilizing the inhibitory synapses. In the *mdx*/bl10 model, the absence of dystrophin disrupts this scaffolding process, leading to a reduction in GABA receptor subunit clustering in multiple brain regions (Knuesel et al., 1999; Vaillend and Billard, 2002; Anderson et al., 2010; Dallérac et al., 2011; Zarrouki et al., 2022a). This leads to weaker inhibitory signalling and a shift in the excitatory/inhibitory signalling balance towards a state of hyperexcitability. As a result, *mdx/*bl10 mice present with altered neuronal firing patterns, impaired synaptic plasticity and behavioural abnormalities (Knuesel et al., 1999; Vaillend and Billard, 2002; Sekiguchi et al., 2009; Anderson et al., 2010; Dallérac et al., 2011; Vaillend and Chaussenot, 2017; Zarrouki et al., 2022a). *Mdx52* mice show even greater alterations in excitatory and inhibitory potentials (Hashimoto et al., 2022). By contrast, Dp71-null mice show alterations in neuronal firing patterns but not in synaptic plasticity (Daoud et al., 2009), highlighting the isoform-specific functions of dystrophin in neuronal signalling. These studies in mouse models are vital, given the inability to measure synaptic activity at a cellular level in humans, making it impossible to directly study the excitatory/inhibitory balance in individuals with DMD. There is, however, indirect evidence, including altered GABA and glutamate levels, and increased seizure risks from magnetic resonance spectroscopy and neuropsychological assessments, that individuals with DMD do present with an excitatory/inhibitory signalling imbalance (Pane et al., 2013; Doorenweerd et al., 2017b).

Arterial spin labelling imaging techniques have revealed that *mdx*/bl10 mice develop reduced cerebral blood flow, as seen in human DMD, but only at an advanced age (>10 months) (Goodnough et al., 2014). Blood–brain barrier permeability is also increased in these mice (Goodnough et al., 2014; Verhaeg et al., 2024), and many proteins related to the blood–brain barrier show altered expression, including ZO-1 (Box 1; encoded by *TJP1*), GFAP (Box 1) and AQP4 (Box 1) (Frigeri et al., 2001; Nico et al., 2003). Blood–brain barrier permeability is also reduced in Dp71-null mice, the loss of which reduces AQP4 clustering (Blake et al., 1999; Daoud et al., 2009; Chaussenot et al., 2019). These changes suggest that dystrophin is not only essential for neuronal signalling but also for cerebrovascular stability and support of the neurovascular system. In mice and humans with DMD, the impaired cerebral perfusion and compromised blood–brain barrier suggest impaired coordination between the neuronal and vascular systems, possibly leading to neuronal vulnerability by the disruption of delivering crucial nutrients and molecules to the cells.

DMD mouse models have made a significant contribution to our understanding of how the lack of dystrophin impacts brain structure, function, metabolism and synaptic signalling. Overall, dystrophin deficiency leads to fear and anxiety, impairments in learning and memory, and disturbances in emotional, depression-related and social behaviour. Furthermore, dystrophin deficiency alters dystrophin cortical and subcortical structures, changes in neurotransmission and the excitatory/inhibitory signalling balance, and leads to cerebrovascular dysfunction. Although interspecies-related differences were identified between DMD mice and individuals with DMD, DMD mouse models are valuable resources allowing more in-depth investigations. A key limitation that warrants attention is the use of steroid-naïve mice, as the majority of individuals with DMD receive corticosteroids as part of the standards of care. Acute and chronic corticosteroid treatment negatively affects behaviour in both humans (Schmidt et al., 1999; Ciriaco et al., 2013; Prado and Crowe, 2019) and WT mice (Gai et al., 2014; Skupio et al., 2015; Dieterich et al., 2019). Chronic corticosteroid treatment exacerbates grey matter volume reductions (Geuens et al., 2023), white matter alterations (Van Der Meulen et al., 2022) and behavioural problems in individuals with DMD (Angelini, 2007; Counterman et al., 2022). Behavioural problems are one of the most common reasons to discontinue treatment (Poysky, 2007; Matthews et al., 2010). Although the use of steroid-naïve animals enables studying the consequences of dystrophinopathy in isolation, it fails to take into account the additive negative effects of corticosteroids on the brain. To what extent chronic corticosteroid treatment affects brain involvement in DMD animal models remains to be investigated. Nevertheless, the value of animal studies lies not in perfect replication of human abnormalities, but in their ability to generate hypotheses, identify target mechanisms and guide therapeutic development, which will be explored in the following section.

## Therapy development for the DMD brain

For many individuals with DMD, as well as for their parents and caretakers, the CNS-related impairments of their disease negatively impact their quality of life (Schwartz et al., 2021, 2022), highlighting the clinical need for CNS-targeting treatment options for DMD. Treatment of the CNS-related impairments consist of management through psycho-education and, occasionally, psychopharmacological therapy to address the psychiatric symptoms of DMD, such as ADHD, ASD and depression (Hendriksen et al., 2016; Lee et al., 2018; Lionarons et al., 2019; Darmahkasih et al., 2020; Noda et al., 2021). Therapeutic approaches that restore dystrophin expression to alleviate muscle-related DMD symptoms have been investigated for decades. These might also be beneficial for treatment of the brain.

Stop-codon readthrough therapy is applicable for nonsense mutations, i.e. pathogenic variants for which a substitution in the DNA converts the code for an amino acid into a premature stop codon. These mutations disrupt dystrophin expression as they lead to premature truncation of protein translation. Chemical compounds (e.g. Translarna) can suppress the premature stop codon, instead facilitating the inclusion of an amino acid and allowing the production of complete dystrophin proteins (reviewed in Politano, 2021). Translarna can cross the blood–brain barrier owing to its hydrophobic nature and was therefore expected to also allow production of brain dystrophin isoforms. Translarna received conditional marketing authorisation for the treatment of eligible individuals with DMD in 2014, pending the collection of additional evidence to confirm reduction of motor function loss. The sponsor failed to collect convincing evidence in two placebo-controlled trials, and, in 2025, the European Medicines Agency (EMA) did not extend the conditional marketing authorization. As such, Translarna will be withdrawn from the market in EU countries. However, it will remain available in the UK, Russia, and several countries in the Middle East and South America (Bello et al., 2025).

Gene therapy, in which the coding sequence of truncated versions of Dp427m (micro-dystrophins, only containing several essential protein domains) is systemically delivered via adeno-associated viral vectors (AAVs), is theoretically applicable to all individuals with DMD. For treatment of the musculature, Elevidys has been approved by the US Food and Drug Administration (FDA) and the Japanese Ministry of Health, Labour and Welfare, based on dystrophin restoration, without proof of functional benefit in the North Star Ambulatory Assessment test in placebo-controlled trials (Mendell et al., 2025; reviewed in Jolly et al., 2025). To date, two cases of acute liver failure have resulted in the death of non-ambulatory patients, which warrants further investigations. Owing to the use of a muscle-specific promotor, and an AAV serotype that does not cross the blood–brain barrier, Elevidys will not result in the expression of micro-dystrophin in the brain.

More recent preclinical developments allow for expression of full-length dystrophins in DMD mouse models (reviewed in Bengtsson et al., 2025), which is achieved by splitting the entire coding sequence over two or three AAVs and using split intein pairs to rejoin the protein products (Tasfaout et al., 2024; Zhou et al., 2024). However, the utilization of muscle-specific promotors and AAVs hampers translatability to the brain.

Gene and base editing, which use CRISPR-Cas9 technologies to directly modify DNA sequences within a cell, either by restoring the reading frame or correcting small mutations permanently, have shown success in the musculature in preclinical DMD models (reviewed in Haque and Yokota, 2025). Several hurdles, such as inefficient delivery to muscle tissue, low editing efficiency, limited treatment durability (owing to muscle turnover and the inability to edit satellite cells) and off-target effects, still need to be overcome.

To date, only the exon skipping approach has been thoroughly investigated in context of dystrophin restoration in the murine brain. This Review, therefore, focuses on this therapeutic approach only.

### Exon skipping to treat the DMD brain

**Fig. 2.**
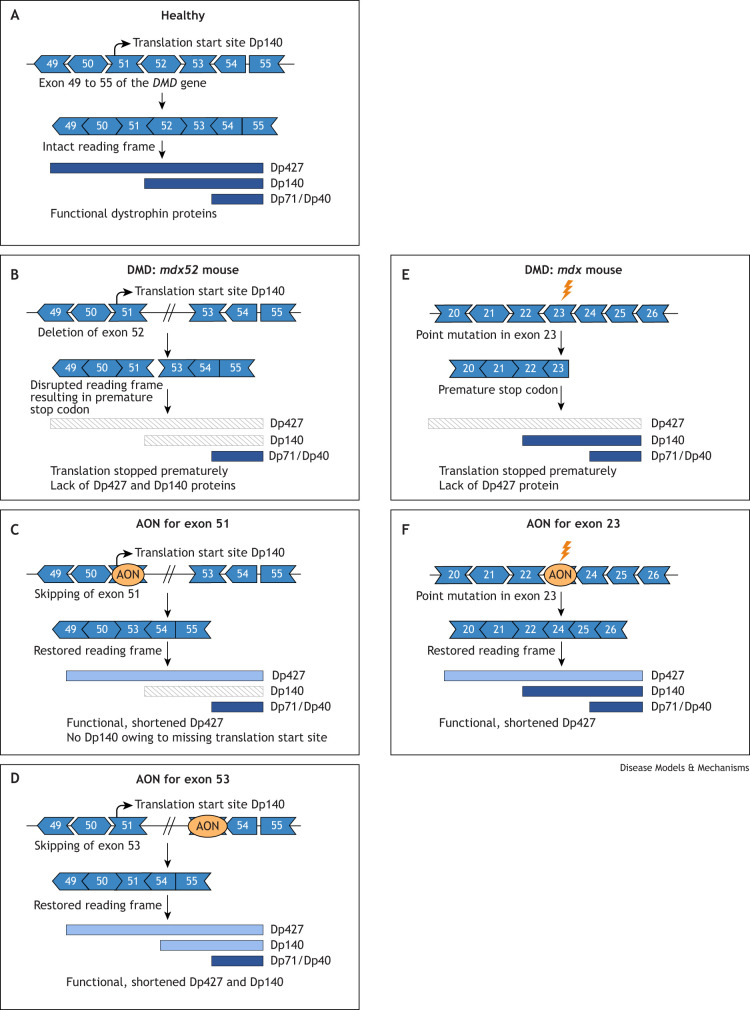
**Exon skipping therapy in DMD.** (A) In a healthy situation, all exons are translated, and dystrophin isoforms are synthesized. (B) In the *mdx52* model, exon 52 of the *Dmd* gene is deleted, which disrupts the reading frame, thereby preventing the synthesis of Dp427 and Dp140, whereas that of Dp71/Dp40 remains unaffected. (C) Skipping of exon 51 restores the disrupted reading frame, thereby allowing the expression of shortened, but partially functional, Dp427 isoforms. Because exon 51 contains the translational start site of Dp140, this isoform is not expressed. (D) When exon 53 is skipped instead, shortened Dp427 and Dp140 proteins are produced. (E) In the *mdx*/bl10 and *mdx*/bl6 models, a point mutation in exon 23 of the *Dmd* gene leads to disruption of the reading frame and a premature stop codon, thereby preventing synthesis of Dp427, whereas the other isoforms remain unaffected. (F) Skipping of exon 23 restores the reading frame and enables the synthesis of a shortened Dp427. AON, antisense oligonucleotide; DMD, Duchene muscular dystrophy.

Exon skipping is a mutation-specific approach that aims to restore the disrupted open reading frame of dystrophin pre-mRNA transcripts by hiding particular exons from the splicing machinery through the utilization of antisense oligonucleotides (AONs; Box 1). Owing to the binding of AONs to the dystrophin pre-mRNA transcript, the targeted exon is spliced out together with its flanking introns. This restores the open reading frame and allows shorter, but partly functional, dystrophin proteins to be translated (Fig. 2).

Depending on the location and extent of a *DMD* mutation, different exons need to be skipped to restore the open reading frame. As most patients have large deletions, involving one or more exons, and these deletions cluster in a hotspot between exons 42 and 55, skipping particular exons is applicable to larger groups of individuals with DMD. AONs that target exon 51 would apply to 14%, those that target exon 45 to 9%, and those that target exon 53 to 10.1%, of individuals with DMD (these numbers are based on the TREAT-NMD DMD Global Database, as reviewed in Bladen et al., 2015). Four uncharged phosphorodiamidate morpholino oligomer (PMO) AONs have been approved by the FDA: Casimersen, which targets exons 45 (Shirley, 2021); Eteplirsen, which targets exon 51 (Mendell et al., 2016; Alfano et al., 2019); and Viltolarsen (Roshmi and Yokota, 2019) and Golodirsen (Frank et al., 2020), which target exon 53. Viltolarsen has also been approved by the Japanese Ministry of Health, Labour and Welfare. These approvals were based on the restoration of low dystrophin levels (1-5%), and it remains uncertain whether these levels can ameliorate disease progression.

Because these uncharged AONs cannot cross the blood–brain barrier, the brain is left untreated. Notably, charged AONs are very efficiently taken up by brain cells when delivered intrathecally. AON-based treatment (Spinraza) has been approved by the FDA and by the EMA for the treatment of all types of spinal muscular atrophy based on compelling data, including reduced risk of mortality and improvements in motor milestones, which are rarely, if ever, achieved in untreated Type 1 spinal muscular atrophy children (Haché et al., 2016; Finkel et al., 2017). For amyotrophic lateral sclerosis, AON treatment (Qalsody) has been approved by the FDA and the EMA based on reduced plasma levels of neurofilament light protein (a marker for neuronal damage), and patients treated earlier performed better – according to an amyotrophic lateral sclerosis functional score – than did those for whom treatment was delayed in open-label extension studies (van Roon-Mom et al., 2023).

The development of exon skipping therapy for brain treatment in individuals with DMD is, however, still in the preclinical phase. Here, we discuss whether the exon skipping approach could also hold promise for the treatment of the brain-related disorders in individuals with DMD. An overview of exon skipping approaches in the murine DMD brain can be found in Table 2, and exon skipping approaches used to restore dystrophin are summarised in Fig. 2.

**Table 2.** Exon skipping approaches in the DMD mouse brain

Mouse strain	Chemistry	Targeted exon	Dose	Route	Duration	Percentage of skipping	Percentage of dystrophin	Behavioural improvement	References
**Restoration of Dp427**									
*Mdx/*bl10	Tc-DNA	23	200 mg/kg weekly	IV	12 weeks	2-4%	Low (not quantified)	Yes (fear response)	Goyenvalle et al., 2015
*Mdx/*bl10	Tc-DNA	23	200 mg/kg weekly	IV	12 weeks	2-5%	5%	Yes (fear response)	Relizani et al., 2017
*Mdx/*bl10	Tc-DNA	23	400 µg	ICV	Single injection	15-35%	5-25%	Yes (at 7 but not 10 weeks after treatment)	Zarrouki et al., 2022b
*Mdx/*bl10	PMO	23	1 mg AON total	ICV infusion with osmotic pump	1 week	25%	25%	Yes (between 5 and 7 weeks after treatment)	Sekiguchi et al., 2009
*Mdx/*bl10	U7-Sd23/BP22 AAV2	23	4.2×10^9^ vg	Intrahippocampal	Single injection	15-25%	25-30%	ND	Dallérac et al., 2011
*Mdx/*bl10	U7-Sd23/BP22 AAV2	23	6.4×10^9^ vg	Intrahippocampal	Single injection	Not quantified	15-25%	GABA_A_ receptor clustering restored	Vaillend et al., 2010
*Mdx52*	Tc-DNA	51	400 µg	ICV	Single injection	20-30%	Not measured	ND	Saoudi et al., 2023a
*Mdx52*	Tc-DNA	51	400 µg	ICV	Single injection	10-15%	5-15%	Yes (anxiety, fear and fear learning)	Saoudi et al., 2023b
*Mdx52*	PMO	51	900 µg per injection	ICV+intra-cisterna magna injections	Every 72 h (4× total)	16-22%	Not measured	ND	Saoudi et al., 2023a
*Mdx52*	AAV9-U7snRNA	51	3E+13 vg	IV	Single injection	5%	0%	No	Aupy et al., 2020
**Restoration of Dp427 and Dp140**									
*Mdx52*	PMO	53	30 mg/kg per injection	ICV	Eight injections over 4 weeks	5-10%	Dp427: 1% Dp140: 5%	Yes (ASD-like behaviour)	Hashimoto et al., 2022
*Mdx52*	PMO (multiple)*	53	400-900 µg	ICV	Single injection	≤25%	Undetectable	ND	Doisy et al., 2023

Exon skipping and its efficiency have been tested in *mdx*/bl10 and *mdx52* mice. In *mdx*/bl10 mice, exon 23 has been targeted, and in *mdx52* mice, exons 51 and 53 have been targeted.

*Three AONs targeting exon 53 being simultaneously delivered.

AAV, adeno-associated viral vector; AON, antisense oligonucleotide; ICV, intracerebroventricular; IV; intravenous, ND, not determined, PMO, phosphorodiamidate morpholino oligomers; Tc-DNA, tricyclo-DNA; vg, vector genome.

#### Restoration of Dp427 expression

The effects of delivering AONs to restore dystrophin expression in the brain were first investigated in the *mdx*/bl10 mouse model. These mice were treated intravenously with tricyclo-DNA (tc-DNA) AONs [15-mer (Box 1) targeting exon 23; Fig. 2E,F] on a weekly basis for 12 weeks (Goyenvalle et al., 2015). Because tc-DNA AONs can cross the blood–brain barrier, this systemic treatment resulted in exon 23 skipping and in the restoration of ∼5% of full-length dystrophin compared to WT dystrophin levels as assessed by western blotting, which led to improvements in the restraint-induced fear response in this model. Comparable results were obtained when shorter 13-mer tc-DNA AONs were used in a similar study setup in *mdx/*bl10 mice (Relizani et al., 2017).

To improve treatment efficacy, a follow-up study treated *mdx*/bl10 mice with a single bilateral intracerebroventricular (ICV) bolus injection of tc-DNA AONs (Zarrouki et al., 2022b). This study reported a dose-dependent increase in exon 23 skipping of up to 35% upon treatment. This, in turn, resulted in a ∼25% increase in dystrophin levels (compared to WT levels) and improvements in the fear response. Dystrophin levels changed over time during this study and were highest 6-7 weeks after treatment (up to 15% of WT levels), and gradually decreased towards week 10. Despite a significant restoration of a normal fear response at 7 weeks, this effect was lost 3 weeks later. Long-term memory retention was also restored in the treated mice, with only minor effects seen for cued fear conditioning.

PMO AONs, another AON chemistry, have also been assessed in *mdx*/bl10 mice, aged 30 days, via ICV infusion with an osmotic pump for 1 week (Sekiguchi et al., 2009). Exon 23 skipping led to restoration of 25% of dystrophin over a time frame similar to that reported for ICV injections with tc-DNA AONs, with optimal restoration seen between 5 and 7 weeks after treatment initiation, and a significant drop in restoration 11 weeks after treatment. The temporal restoration of dystrophin was mirrored by partial rescue of the freezing response during the 5- to 7-week post-treatment window.

To optimize treatment efficacy, several delivery routes have been tested for tc-DNA and PMOs in the *mdx52* model in which skipping of exon 51 restores expression of Dp427 (Fig. 2B,C) (Saoudi et al., 2023a). ICV injections of tc-DNA resulted in the highest exon 51 skipping levels (20-30%), while a combination of ICV and intra-cisterna magna injections (Box 1) yielded the best results for PMOs (16-22% exon 51 skipping). In addition, a single bilateral ICV was found to be more efficient than unilateral injections at exon skipping, while slow delivery increased the distribution of AONs compared to rapid injection, although skipping levels were similar regardless of the delivery speed. Repeated ICV injections did not increase exon skipping levels (relative to single ICV delivery).

Although in the described studies the levels of restored dystrophin remained relatively low, the therapeutic-relevant responses that were produced were promising. Saoudi et al. (2023b) showed that 10-15% of exon skipping via a single ICV injection of tc-DNA led to 5-15% of Dp427 restoration in *mdx52* mice, which was enough to reduce anxiety and unconditioned fear, improve fear memory and completely rescue fear conditioning.

Exon skipping can also be induced by using U7 small nuclear RNAs. These RNAs encode antisense sequences that can target a particular exon and are expressed from recombinant AAVs (Goyenvalle et al., 2004). Single intra-hippocampal injections of U7-Sd23/BP22 AAV2 vectors partially restored expression of Dp427 in the hippocampus (∼25-30%) in *mdx*/bl10 mice, which lasted for at least 2 months. This led to completely recovered GABA_A_-receptor clustering and hippocampal synaptic plasticity in the mice (Dallérac et al., 2011). This response has been replicated in another study in which *mdx*/bl10 mice were treated with a slightly higher dose of the same vector, restoring 15-25% of Dp427, which recovered GABA_A_-receptor clustering for up to 4 months (Vaillend et al., 2010). Systemic treatment of *mdx52* mice with an AAV serotype 9 (AAV9)-U7 targeting exon 51 vector induced ∼5% exon 51 skipping in the cortex, hippocampus and cerebellum of these mice, which was too little to restore dystrophin protein and address the behavioural deficits (Aupy et al., 2020).

#### Restoration of Dp140 expression

Dp140 is primarily expressed in the developing human brain (Doorenweerd et al., 2017c; Catapano et al., 2025). It is therefore unclear whether its postnatal restoration would have any therapeutically relevant effect. This question has been addressed in the *mdx52* mouse model in which skipping of exon 53 not only restores Dp427 but also Dp140 expression (Table 2, Fig. 2B,D). These mice were treated with eight ICV injections, with PMOs targeting exon 53, over 4 weeks, which resulted in ∼5-10% skipping of exon 53 and the restoration of 1% of Dp427 and 5% of Dp140 (Hashimoto et al., 2022). Although dystrophin restoration was very low, ASD-like behaviour and the alterations in excitatory/inhibitory signalling balance were ameliorated in treated animals. No differences were found in their anxiety or fear response relative to that of untreated controls.

A multicentre study using different AON chemistries has since tried to improve exon skipping and dystrophin restoration levels (Doisy et al., 2023). A combination of three AONs was required to improve exon 53 skipping to 25% (in the hippocampus), regardless of the chemistry used, highlighting the challenges of exon 53 skipping compared to exon 51 skipping in *mdx52* mice.

## Challenges and future directions

Thirty percent of individuals with DMD experience behavioural and cognitive deficits that impact their quality of life and ability to integrate into society. These issues highlight the clinical need to develop therapeutics that can provide effective treatment of these conditions to ameliorate them.

Despite the knowledge gained to date, there is still a lot to learn about CNS involvement in DMD. Clinical studies are restricted by small sample sizes and lack of adequate representation of individuals with more distal mutations affecting expression of multiple or all dystrophin isoforms (which is a direct consequence of the lower prevalence of these mutations). Studies on brain involvement in DMD animal models have filled this gap to some extent. Linking the neurobehavioural observations between humans and mice, however, remains an important challenge. To improve translatability of preclinical findings to the clinic, future preclinical studies could consider including DMD and WT mice chronically treated with corticosteroids. Given the suspected negative contribution of chronic corticosteroid use on DMD brain involvement in individuals with DMD, more in-depth investigation in this direction in animal models is needed.

Despite the encouraging preclinical data obtained with the exon skipping approach to treat the murine DMD brain, our knowledge is incomplete, with practical challenges and theoretical questions limiting translation of this approach to the clinic. First, restoring expression of the low-abundant *Dmd* transcript in the brain appeared especially challenging when targeting exon 53 in the *mdx52* strain. Three AONs were required to do so, whereas efficient exon 51 skipping only required a single AON. This is in sharp contrast to the human *DMD* gene, in which exon 53 seems easier to skip than exon 51 (Doisy et al., 2023). This discrepancy could result from differences in transcript processing dynamics between mice and humans (Gazzoli et al., 2016; Spitali et al., 2013). The use of appropriate humanized mouse models could increase translatability of future preclinical studies by direct evaluation of human-specific sequences. Exemplary here could be the hDMDdel52/*mdx* mouse, which carries the entire human *DMD* gene with a partial deletion of exon 52 that prevents expression of Dp427 and Dp140 (Veltrop et al., 2018; Yavas et al., 2020). When crossed with *DMD-null* mice, expression of all murine brain dystrophin isoforms could be eliminated.

Second, repeated treatment is required to maintain dystrophin expression because AONs target the pre-mRNA. Investigations on the effects on CNS impairments have predominantly utilized tests with a short execution time and a large therapeutic window, such as the unconditioned fear test. Consequently, the field still lacks a complete picture of the full potential of postnatal dystrophin restoration. In the future, it might be interesting to focus on investigations of other behavioural domains or the suitability of imaging techniques (e.g. MRI) as outcome measures.

Third, in light of the non-progressive nature of the CNS involvement and the unique spatio-temporal expression profiles of each of the brain-specific dystrophin isoforms, with Dp427p and Dp140 being predominantly expressed before birth (Doorenweerd et al., 2017c; Catapano et al., 2025), it seems likely that these isoforms play a role in neurodevelopment. Postnatal restoration of these isoforms may therefore not be able to reverse any of the neurodevelopmental deficits. Contrastingly, postnatal restoration of the isoforms expressed throughout life, such as Dp427c, could be more likely to be therapeutic. To what extent postnatal restoration of Dp427 or Dp140 holds therapeutic potential remains to be investigated.

The discoveries and developments discussed in this Review have significantly increased our understanding of CNS involvement in DMD. The impact of this research is not limited to DMD but could help us to better understand the biology of DMD comorbidities such as ASD and ADHD. Further in-depth investigation of molecular and cellular pathways and their influence on brain plasticity and function in DMD models could also potentially shed light on therapeutic targets, which might, in addition to DMD, hold promise for this broader spectrum of neurodevelopmental disorders.
